# Severe cognitive disability, medically complex children and long-term ventilation

**DOI:** 10.1007/s40592-025-00234-5

**Published:** 2025-02-26

**Authors:** Helen Turnham, Dominic Wilkinson

**Affiliations:** 1 Consultant Paediatric Critical Care, Oxford University Hospitals NHS Trust, John Radcliffe Hospital, Headley Way, Oxford OX3 9DU, UK; 2 John Radcliffe Hospital, Neonatal medicine, Oxford, UK; 3 Murdoch Children’s Research Institute, Melbourne, Australia; 4 Centre for Biomedical Ethics, National University of Singapore Yong Loo Lin School of Medicine, Singapore, Singapore; 5 Department of Paediatrics, Faculty of Medicine Nursing and Health Sciences, School of Clinical Sciences, Monash University, Melbourne, Australia; 6 Oxford Uehiro Centre for Practical Ethics, Faculty of Philosophy, University of Oxford, Suite 1 Littlegate House, St Ebbe’s Street, Oxford OX1 1PT, UK

**Keywords:** Cognitive disability, Long-term ventilation, Ethics

## Abstract

Children with complex medical conditions including those with severe intellectual disability are living longer. For some, support with medical technology such as Long-Term Ventilation can prolong their lives further. Such technological supports can have significant implications for the child and her family and consume considerable resources though they can also offer real benefits. Sometimes clinicians question whether children with very severe cognitive impairments should have their life prolonged by technology, though they would be prepared to provide the same treatment in equivalent cases without cognitive disability. We describe and analyse four ways in which this view might be justified. Although it could be claimed that children with severe cognitive disability have lives that are not worth living, in most cases this view can and should be rejected. However, the burdens of life-prolonging technology may outweigh the benefits of such treatment either in the present or in the future. Consequently it might not be in their interests to provide such technology, or to ensure that it is provided as part of a time-limited trial. We also consider circumstances where medical technology could offer modest benefits to an individual, but resources are scarce. In the face of resource imitation, treatment may be prioritised to children who stand to benefit the most. This may in some circumstances, justify selectively withholding treatment from some medically complex children.

## 1 Introduction

Children with complex medical conditions are living longer. Young people who once might not have been expected to survive infancy can now live to be adolescents and adults. For some children, living a longer life is facilitated by medical technology. Recent advances have allowed treatments that were once confined to the intensive care be delivered at home and for long periods (years). For example; mechanical ventilation through a tracheostomy, renal replacement therapy and even mechanical hearts. But such treatments are potentially burdensome for the child and caregivers and consume limited resources. In this paper, we will focus on Long term ventilation (LTV), though the ethical questions overlap with other forms of life-prolonging technology.

There are many factors that must be weighed up in deciding whether to provide LTV. However, in our experience, one influential factor in discussions and deliberation is the presence or absence of cognitive disability.

Consider the following two children, Ali and Alison, both 10 years old and receiving acute treatment in the paediatric critical care. (These are composite cases, representative of the authors’ experience).

## 2 Cases and key question

### 2.1 Ali

Ali inherited a form of muscular dystrophy which causes progressive weakness of his muscles. He was diagnosed as a toddler, over time Ali has lost muscle strength, he is now dependent upon a wheelchair and needs assistance with many physical tasks. He attends school and is noted to be gifted at maths and languages and has won a prestigious scholarship to an academically competitive school.

Ali was admitted into the paediatric critical care 4 weeks ago with a lower respiratory tract infection. He is dependent upon invasive ventilation because his respiratory muscles have become too weak to support breathing independently. His medical team consider if he should have a tracheostomy formed and to go home with long term ventilation.

It is possible that he could live at home for (many) years with support from the ventilator, though the nature of his illness is life-limiting. Without ventilation he is likely to die within days to weeks. If he undertakes long term ventilation this is, likely to be required for the rest of his life; there is little chance that he would ever be able to live independent of the ventilator.

### 2.2 Alison

Alison suffered a brain injury during her birth that has left her with cerebral palsy. She has a significant cognitive disability [her abilities can be illustrated as being akin to those of a young infant]. She uses a wheelchair with a head support, relies on others for all activities of daily living such as moving her limbs, clearing secretions, feeding, and turning over in bed. Alison is unable to speak, and she is fed through a gastrostomy. She suffers seizures that are well controlled with medication. Alison expresses pleasure (vocalisations and head movements) when her siblings play around her and particularly when there is lively music and sensory lights.

Alison was admitted to paediatric critical care six weeks ago with a lower respiratory tract infection. She has failed several attempts to wean from ventilation due to a combination of poor central drive to breathe (brain capacity to stimulate a breath), poor cough and copious oral and respiratory secretions. Her medical team consider if she should have a tracheostomy formed to facilitate long term ventilation.

It is possible she could live at home for (many) years with support of a ventilator, without ventilation she is likely to die within days to weeks. If she undertakes long term ventilation this is likely to be required for the rest of her life; there is little chance that she will be able to live independently of the ventilator.

Reflecting on these two cases as we have described them, both Ali and Alison could live for several more years into young adulthood with LTV but their life expectancy is uncertain. A key difference between Ali and Alison is that Alison is cognitively disabled, while Ali is not. To our knowledge, there are no empirical reports describing the relative weight of different ethical considerations in decisions about LTV. However, in our experience, the presence or absence of cognitive disability has a major influence on decisions. To put it bluntly, for children who are ventilator dependent but cognitively able, there is often a sense that LTV is ethically appropriate and possibly even mandatory to offer. In contrast, for children with equivalent degrees of respiratory failure and life expectancy but who have severe cognitive disability there is often reluctance to offer LTV, and sometimes a sense that this would be ethically wrong to provide. Framed in this way, the key question for this paper is whether it is justified or alternatively whether it would be discriminatory to treat Alison differently from Ali.

Making the decision to support a child with ventilation for the duration of their life, ie as a so-called “destination therapy” is important as the consequences of therapy or not supporting with ventilation and thus allowing a child to die, are profound.

## 3 Consequences of long term ventilation

### 3.1 Living with a tracheostomy and LTV

The technology required to live with a ventilator is relatively simple, ventilators are now small, transportable, simple to maintain and relatively cheap to provide. There are many other sorts of equipment required including oxygen, suction devices and humidifiers to name a few. But many adults and children successfully live longer lives supported by invasive ventilation.

In general tracheostomies are comfortable to live with and whilst suction of secretions can be uncomfortable it is not usually significantly painful when performed by experts. Children and young people need a lot of additional care, such as daily chest physiotherapy and delivery of nebulised drugs. Some children can learn to swallow and thus eat and talk when they have a tracheostomy, but not all children. For many there is a loss of eating food and speaking and smell.

Children and young people who live with a tracheostomy have a constant background risk to their lives (McDougall 2013). This requires that they have round the clock specialist care. In the event of a sudden tracheostomy blockage or misplacement, the child can develop dangerously low oxygen levels and very quickly die.

In some jurisdictions care is delivered in long term care facilities, in others home care is default and parents undertake specialised training to care for their children including learning to suction through a tracheostomy, how to change a tracheostomy in an emergency and how to deliver oxygen. It is usual for children to always require two trained adults with them, including one adult who is awake and monitoring the child night and day. Parents cannot deliver this intensity of care continuously for their children alone and an extensive package of care is developed to support the child and family, delivered by nurses or trained care workers.

Such complex care may take many months to organise, up to eighteen months in our tertiary childrens hospital. Whilst waiting for staff to be recruited and trained, children and their families live in hospital. Life is extremely difficult for the young person and their family (Turnham et al. 2024).

Parents who care for children supported with technology, can find their roles substantially altered. They often stop work outside the home and become full time carers, siblings of the child become young carers. Whilst families can and do deliver expert and excellent care for their children the psycho-social impact on them is high, particularly for mothers (Boettcher 2020) who “experience high levels of depression, burnout, burden and low levels of self-esteem” The siblings are also affected as adult responsibilities are transferred to the well sibling, and well siblings are often left in the shadow (Alrø 2021).

### 3.2 Dying with a tracheostomy and LTV

Long term ventilation can overcome decline in drive to breath and cough adequacy as well as muscle weakness leading to respiratory failure. But children with degenerative diseases such as Ali will continue to decline, Ali will get weaker and his heart may fail. Even children who have static brain injuries, such as Alison has experienced can develop failure of other organ systems over time.

Long term ventilation can only overcome poor drive to breath, weak cough and weak respiratory muscles. Decline in other organ systems for Alison might include the brain with uncontrolled seizures or dystonia (a painful and uncontrolled contraction of the muscles). She might develop gastrointestinal failure with feed intolerance, chronic vomiting, severe constipation and bowel obstruction. Symptoms from these might be hard to manage and a child may experience a difficult death.

### 3.3 Provision of long-term ventilation

Living with long term ventilation requires substantial resources, both in terms of equipment and personal. Typical packages of nurse care at home, or in specialised units cost hundreds of thousands of dollars per year. Home ventilation requires the availability of nurses or carers with an extended and specialist set of skills. There are a limited number of persons trained and willing to be trained to deliver this high-risk care. There is a rapidly increasing population of children requiring highly specialised home care (Barker 2023). It is possible that the ability to provide LTV becomes limited by the resources available.

## 4 Making decisions about long-term ventilation for children

Some children might be able to express preferences about proposed medical treatments, but consent or refusal is beyond the developmental and experiential capabilities of such young children as Ali and Alison and most jurisdictions would not legally permit a young child to make this decision. Thus, they require surrogates to make decisions for them.

Best interests are the foremost principle when considering medical treatments for children. Parents, who are the usual decision-makers must act to promote the interest of their child, this might include taking into account the preferences of their child if they can make and communicate them. The moral argument for parents being usual decision-makers is laid out in Turnham et al. (2020). In turn, medical professionals should only offer treatments that are in the child’s interests.

For both Ali and Alison, lifelong ventilation offers the opportunity of a longer life. Without ventilation is it likely that both will die shortly. There is, by necessity, a high ethical bar to be met before it is determined to be against a child’s best interests to live a longer life.

For Ali, living with lifelong ventilation allows him to continue his studies, attend school, make friends. But with some new restrictions, he will depend upon greater care than previously; likely endure a lengthy hospital admission. There would need for suction of his respiratory secretions, he may lose his ability to eat, smell or talk.

Alison is also likely to live a longer life with lifelong ventilation. She might have improvements in the quality of her life, for example a reduced number of admissions to hospital than before, respiratory secretions that are easier to manage (suction through a tracheostomy is more comfortable than suction through the mouth or nose that she required before). Alison may have better sleep quality and be more alert during the daytime.

Individuals are likely to have different preferences as to the benefits and burdens offered by destination treatment with long term ventilation. *But* for many, the benefits of a longer life will justify the burdens of living with ventilation.

### 4.1 Is the presence of severe cognitive disability ethically relevant to decisions about technological support for medically complex children?

Whilst Alison may have benefit from life-long ventilation, the benefits are objectively more modest than those Ali might enjoy. Her severe cognitive disability means that she will never be able to build independent relationships with others and she will rely upon others for all activities of daily living.

A longer life for Ali gives him an opportunity to live a life that looks like that which we hope for every child and ourselves. That is, a life with potential to develop his own interests and preferences, build independent relationships, to study and perhaps one day a job. The benefits that Ali stands to gain are so great that some might regard it as morally unacceptable *not* to prolong his life with LTV. In contrast, some might wonder if Alison should live a longer life supported with ventilation.

We consider four ways this view might be justified;

- A life that is not worth living.
- A life that is worth living but not with the burdens of mechanical ventilation.
- A life that is worth living in the short term if provided with tracheostomy/LTV but the risk of an unbearable future (including of a bad death) justifies with-holding treatment now
- A life that is worth living if resources are available, but if resources are scarce, it might be justified to prioritise other patients for this support.

#### 4.1.1 A life not worth living

Some might consider that due to her significant cognitive impairment Alison’s life (even prior to her developing a severe chest infection) is not worth living. In a different case, where Alison suffers continuous seizures or intractable dystonia such that she is in pain or distress most of the time, such a view may be defended. It might not be in Alison’s interests to provide any life-prolonging treatment. However, if that were the case, it would have been wrong to initiate intensive care six weeks ago. Moreover, it would imply that it would be wrong to provide any form of life-prolonging treatment. Alternatively, if Alison had suffered a new brain injury (perhaps as a consequence of her acute illness), that she was largely or entirely unaware of her surroundings, it might be a reasonable view that we should not prolong her life with ventilation. But it is not reasonable to conclude that Alison’s life is not worth living just because she has a significant cognitive disability.

Consider, for a moment, the considerable moral difficulty when defining personhood or self (used interchangeably here) as there are similarities with our discussion. Personhood is both descriptive (to be a self, one must fulfil descriptive conditions) and normative (being a self comes with a superior moral status). Most definitions require cognitive abilities, and this leads to unintended “normative implications” (Leuenberger 2023) that individuals who we would “like to see treated as persons do not meet [their] criteria for personhood because they do not have certain cognitive capacities”. We risk a similar charge if we consider that severe cognitive impairment renders Alisons life is not worth living and not worth prolonging with technology.

A Pattern Theory of Self (PTS) (Gallager 2013) offers a solution to overly narrow definitions of personhood and illustrates how Alison and Ali are different but with one no less a person than the other. PTS unifies multiple descriptive conditions of self. Gallagher’s non-exhaustive list includes narration, embodied elements, minimal experiential, affective, behavioural, inter-subjective, cognitive, reflective, normative, situational. In PTS no single description has greatest value, the relative importance for each is fluid and differ between individuals who share equal moral status as persons.

Ali, as for many (Leuenberger 2022), narration has special significance because it allows for self-definition. Alison does not narrate, but she is equally a person. For Alison, situational consideration might be of greater importance, for example the very special relationship families have with their disabled children. It is important that those who narrate (in this case those of us who contribute to the decisions made for Alison), are not biased when considering the life that Alison experiences. Medical teams working in the acute setting of intensive care are unlikely to have seen Alison when she is well and to have not observed the areas of her life that best hold value and quality for her and for her parents/guardians. “Clinicians need to be aware of their own biases when making decisions regarding ventilation” (Klee et al. 2022). It is important for such significant decisions to be shared with the long term clinicians and allied health professionals who care for her long term and might have more insight as to the quality of her life when well.

#### 4.1.2 Burdens of long-term ventilation

Previously we have discussed what life looks like for those living with LTV because this is a treatment that has significant implications. Alison appears to have a life that has been worth living before her admission to hospital and this would continue if she were able to return to a life that resembles this prior life. But if LTV were to change her experience of life very significantly, the burdens of LTV may outweigh the modest benefit of returning to her prior life.

For example, if long term ventilation were to commit Alison to living a prolonged period (perhaps the rest of her life) in a hospital intensive care, without home comforts and without the constant presence of her loving family and no chance of living the life that held significant value for her, it might be that the burdens of long term ventilation exceed the modest benefits and we could be justified in not prolonging her life with LTV. Similarly, if long term ventilation were to mean that Alison were unable to leave her home or enjoy the things that had previously given her pleasure, that might mean make LTV contrary to her interests.

If the concern is about the negative effect of tracheostomy and LTV on Alison’s quality of life, that might be dependent on the support (including symptom management) provided to her. Whether this argument applies may depend on the social, nursing and medical support that is available to Alison and her family in the event of LTV being provided. It may mean that in some settings LTV would not be in her interests (for example, where she will have prolonged hospitalisation and/or a lack of supportive specialist care at home), but that in other settings it would be.

#### 4.1.3 Risk of an unbearable future life

Alison might live longer, supported by long term ventilation and her life could be meaningful for her and her family. However, if the future consequences to her were so burdensome, we might justify not prolonging her life with LTV.

For example, we have outlined limitations of LTV to overcome respiratory failure but not prevent decline in other systems. Children with life limiting diseases will continue to decline and some children with static brain injuries also decline over-time. Development of organ failures such as feed intolerance or intractable seizures can be hard to manage at the end of life and for some, result in life and a death that is painful or distressing.

One very significant challenge in practice is discontinuing long-term ventilation once initiated. Alison will always require others to notice that she is no longer benefiting from a longer life. (Ali is protected to some extend from these burdens as he can clearly communicate his own discomfort and in time even make his own decision to stop treatment.) There is risk that, because of their love for her, or because of their personal values or religious views, Alison’s family will be unable to stop invasive ventilation at a future point if it were no longer benefiting her. Additionally, whilst is established that there is no moral distinction between withdrawing and withholding medical treatment (Reichen 2014) medical teams find withdrawal of life sustaining treatment more psychologically and ethically problematic that withholding treatment (Chung 2016). These factors combined may mean that Alison suffers a possibly prolonged period of treatment that is contrary to her interests. Particularly if the symptoms experienced at end of life are not related to ventilation, such as in the situation of acute on chronic bowel obstruction causing intractable vomiting, this is a symptoms difficult to palliate effectively.

However, if the concern is about Alison’s future wellbeing and the difficulty of withdrawing LTV once started, the solution may be to provide treatment in the form of a time-limited trial (Kruser 2024), and to provide support for both Alison and her care team to review the benefits of treatment at ongoing intervals, and to make timely decisions to withdraw if or when that is in her best interests.

#### 4.1.4 Directing scarce treatments to those that benefit the most

If, with LTV Alison could live a longer life that is meaningful, and she is mostly comfortable both now and, in the future, there is a strong prima facie argument that Alison should be offered long term ventilation.

However, each child receiving LTV support requires a package of nursing care that costs hundreds of thousands of dollars per year. In many countries, the amount of funding available to support highly expensive and resource intensive treatments is limited. Significantly it is increasingly difficult to recruit nurses who can provide care and assistance for children with LTV at home. Children face longer and longer periods living in acute hospitals as there are not enough carers available to look after them at home. Furthermore, children supported with LTV may require prolonged initial admission and then frequent require readmission to paediatric intensive care with intercurrent illness. Given scarcity of PICU beds, provision of LTV can negatively impact on the ability of critical care services to support other children.

Where resources are scarce there is a moral imperative to ensure that those that can benefit most from treatment receive it. The ethical principles underlying this are the same as those that apply to other resource limited therapies such as organ transplantation or extra corporeal membrane oxygenation when maximising benefit is considered (Persad 2009).

If there is a limit to the number of children who can be supported with long term ventilation, it is vital that there is a transparent, consistent and robust process for allocation that is free of bias, and carefully considers all morally important considerations. (For example, this might include national or regional allocation committees, and/or a set of allocation factors, analogous to those used for transplant listing). It is also important to note that if LTV were declined on the grounds of inadequate resources, this should potentially mean there would not be a reason to decline treatment if resources were to be available from other sources with no impact on delivery of care to others (for example private financing of treatment or extended family training to deliver care) it would be morally acceptable to facilitate that therapy according to the interests of Alison.

## 5 Conclusion

Ali and Alison will die soon unless their lives are prolonged by LTV. Arguably, whilst there are benefits to both, for Ali the benefit is more substantial than the modest benefit to Alison.

Alison has a life worth living, notably revealed through her special relationship with her family, who share her best days. LTV could offer her the opportunity to continue life resembling her preadmission life and with some modest additional benefit, such as suction of secretions that is more comfortable. There is no suggestion, from what we know, that her life is painful or distressing thus it might be reasonable to offer her support with LTV if there are resources available. But, given the current and possible future burdens with, at best, modest benefit, it would also be reasonable to not initiate LTV. The decision therefore falls within a zone of parental discretion and depends on her parents’ preferences.

Ali will have substantial benefits with a longer life albeit there are new burdens for Ali and his life will be very different to his peers, but with support he could develop his own relationships outside those of his immediate family, attend school to continue his studies and develop the capacity to make his own decisions. Potentially, including the decision to continue or stop treatment for himself in the future. The benefits to Ali are such that the threshold might be reached where it could be morally harmful not to support him with LTV and the decision fall outside the zone of parental discretion.

If there were a limit to the numbers of children who could be supported with LTV directing resources toward those who benefit the most is morally important. This might mean that Ali is offered LTV but Alison is not.

## Data Availability

No datasets were generated or analysed during the current study.

## References

[R1] Alrø AB, Høyer L, Dreyer P (2021). A child with home mechanical ventilation affects the family: A Danish study shows that well siblings may become shadow children. Journal of Pediatric Nursing.

[R2] Barker N, Sinha A, Jesson C, Doctor T, Narayan O, Elphick HE (2023). Changes in UK paediatric long-term ventilation practice over 10 years. Archives of Disease in Childhood.

[R3] Boettcher J, Denecke J, Barkmann C, Wiegand-Grefe S (2020). Quality of life and mental health in mothers and fathers caring for children and adolescents with rare diseases requiring long-term mechanical ventilation. International Journal of Environmental Research and Public Health.

[R4] Chung GS, Yoon JD, Rasinski KA, Curlin FA (2016). US physicians’ opinions about distinctions between withdrawing and withholding life-sustaining treatment. Journal of Religion and Health.

[R5] Gallagher S (2013). A pattern theory of self. Frontiers in Human Neuroscience.

[R6] Klee K, Wilfond B, Thomas K, Ridling D (2022). Conflicts between parents and clinicians: Tracheotomy decisions and clinical bioethics consultation. Nursing Ethics.

[R7] Kruser JM, Ashana DC, Courtright KR, Kross EK, Neville TH, Rubin E, Schenker Y, Sullivan DR, Thornton JD, Viglianti EM, Costa DK (2024). Defining the time-limited trial for patients with critical illness: An official american thoracic society workshop report. Annals of the American Thoracic Society.

[R8] Leuenberger M Who gets to be a person? | Practical Ethics (ox.ac.uk).

[R9] Leuenberger m (2023). A Narrative Pattern-Theory of the Self in Personhood, Self-Consciousness, and the First-Person Perspective, Hermann.

[R10] McDougall CM, Adderley RJ, Wensley DF, Seear MD (2013). Long-term ventilation in children: Longitudinal trends and outcomes. Archives of Disease in Childhood.

[R11] Persad G, Wertheimer A, Emanuel EJ (2009). Principles for allocation of scarce medical interventions. Lancet.

[R12] Reichlin M (2014). On the ethics of withholding and withdrawing medical treatment. Multidisciplinary Respiratory Medicine.

[R13] Turnham HL, Binik A, Wilkinson D (2020). Minority report: can minor parents refuse treatment for their child?. Journal of Medical Ethics.

[R14] Turnham HL, Bowen SJ, Ramdas S, Smith A, Wilkinson D, Harrop E (2024). As low as reasonably practicable (ALARP): A moral model for clinical risk management in the setting of technology dependence. Journal of Medical Ethics.

